# Floor vibrations for motivation and feedback in the rat vibration actuating search task

**DOI:** 10.1371/journal.pone.0257980

**Published:** 2021-09-27

**Authors:** Darian Lawrence-Sidebottom, Michelle A. Schmidt, Daniel O. Harvey, Hans P. A. Van Dongen, Christopher J. Davis

**Affiliations:** Elson S. Floyd College of Medicine and Sleep and Performance Research Center, Washington State University, Spokane, WA, United States of America; Tokai University, JAPAN

## Abstract

Motivating rodents to perform cognitive tasks often relies on the application of aversive stimuli. The Vibration Actuating Search Task (VAST) is a novel open-field task in which gradient floor vibration provides motivation for the rodent to navigate in the direction of diminishing vibration to an unmarked target destination. Using floor vibration as a motivational stimulus may overcome several of the potential confounds associated with stimuli used in other tasks. In a series of three experiments, we determined whether (1) rats exhibit place preference for floor vibration over other aversive stimuli (i.e., water, foot shock, and bright light), (2) exposure to floor vibration is associated with a lower corticosterone response than exposure to these other stimuli, (3) rats successfully acquire the VAST, and (4) VAST performance is sensitive to 6 h of sleep deprivation (SD). Our results showed that rats exhibited place preference for vibration over water, foot shock, and bright light environments, and that corticosterone levels were lower in rats exposed to vibration than those exposed to water. VAST performance also significantly improved over two days of testing for some metrics, and SD impaired VAST performance. Overall, we conclude that (1) rats exhibit place preference for vibration over other stimuli commonly used to motivate task performance, (2) the vibrations employed by the VAST produce lower concentrations of circulating corticosterone than forced swimming, (3) rats can learn to use gradient floor vibration as a mode of performance feedback within two days of testing, and (4) VAST performance is substantially impaired by SD. Thus, the VAST is an effective and practical testbed for studying the mechanisms by which SD causes deficits in feedback-dependent decision making.

## 1. Introduction

Assessing cognitive performance in rodent models was pioneered at the end of the 19th century in the form of intricate mazes [1]. Since then, rodent task designs and applications have evolved and now comprise a myriad of behavioral approaches that are popular tools in the fields of psychology, neuroscience, pharmacology, and biomedicine. A primary consideration in the selection and interpretation of rodent task performance models is motivation. Incentives to boost motivation are typically rooted in a hedonistic spectrum that ranges from appetitive to aversive stimuli.

Performance incentives based on an appetitive reward, such as providing access to food pellets or sucrose solution to reinforce task-related behavioral responses, as seen in many maze and operant learning paradigms, are confounded by satiation, which reduces reward salience. Therefore, rodents are commonly food- or water-restricted prior to task performance to increase the motivational salience of an appetitive reward and counteract satiation. However, the use of appetitive rewards with food or water restriction introduces potential confounders to performance testing, including changes to the animal’s physiological state and a gradual decrease in motivation with food or water intake across the task duration [2].

On the other hand, performance incentives based on aversive stimuli, including forced swimming, bright light, and foot shock, are confounded by the induction of stress, which can affect behavior and thereby impact cognitive performance testing. In the Morris water maze, a rodent visuospatial navigation task [3] that has been used as a learning and memory assay, rodents are placed in a round tub of water and must swim until they locate a submerged platform. Rodents exposed to the task encode peripheral visual cues to aid faster and more direct escapes from the water. However, the paradigm introduces experimental confounds such as fatigue from swimming and physiological effects of thermoregulation. Moreover, the water involved in the task introduces logistical and technical challenges, including room accommodations for draining and modifications needed for electrophysiological recordings in the presence of water.

In the Barnes maze task, bright overhead lights motivate performance based on rodents’ instinctive behavior to escape from bright light environments [4]. The task paradigm is composed of an elevated wall-less circular platform with 18 to 20 outer edge holes that are equidistant from one another and from the center of the maze. An escape box is located below one of the holes, and the rodent uses visual cues to learn to locate the hole with the escape box. In both the Morris water maze and the Barnes maze, cortico-hippocampal networks are necessary for task acquisition, and these platforms can be valuable for assessing specific neurobehavioral circuits [5, 6]. Although lower intensity maze light has been used [7], issues caused by the use of aversive stimuli may inadvertently produce behavioral and physiological confounds associated with stress in these paradigms [8].

Fear conditioning is a different type of paradigm that is widely used to motivate behavior. This classical conditioning paradigm pairs an aversive unconditioned stimulus, such as a foot shock, with an environment (contextual) or a tone or other (cued) conditioned stimulus. Thus, fear conditioning paradigms also introduce stress, which can be a valuable tool in some types of research, including work on post-traumatic stress. However, the behavioral and physiological changes associated with stress (e.g., motionlessness, increases in corticosterone) can be problematic when stress is not the intended experimental manipulation.

To address the potential confounds associated with behavior motivated by any of the aforementioned methods, we developed a performance task called the Vibration Actuating Search Task (VAST). This task, which was first introduced by our group as a mouse paradigm [9], employs a vibration stimulus that is expected to be less aversive and less stressful than water, bright light, or foot shock environments. Here, we assess the VAST as a rat task platform.

In the VAST, a floor vibration gradient provides motivation for the rodent to navigate in the direction of diminishing vibration to where an unmarked target destination is located. Although still and quiet environments over a vibration setting are preferred, floor vibration is not overtly noxious or life-threatening to rats. Therefore, the stimulus employed by the VAST is less likely to induce unwanted confounds associated with stress. The VAST may also resolve issues that accompany the use of water in the Morris water maze, such as fatigue from swimming or thermoregulation challenges. In addition, rats respond to floor vibration gradients naturally [10], requiring minimal preconditioning, so the VAST may obviate the weeks-long training regimen necessary for shaping and maintaining operant behaviors in many other rodent performance paradigms.

The VAST is conceptually based on the children’s game “hot and cold,” wherein the search for a hidden object is cued with verbal feedback as to the proximity to the target. In the VAST, however, the object is replaced by an unmarked target destination, which changes in location from trial to trial, within an open field. At the beginning of each trial, the rat is placed in the open field at one of four locations. Floor vibrations then begin, and changes in frequency are based on the rodent’s proximity to the target location. As the rat moves toward the target, the vibrations become slower, and as the rodent moves away from the target, the vibrations become faster. Thus, the rat’s distance from the target destination modulates the stimulus frequency in a smooth, graded fashion. Upon arrival at the target location (or after 120 s if the target location is not found), the vibrations cease, and the rat is removed from the open field. During each trial, the rat’s movements are tracked with infrared camera technology. Target finding success rate, path distance, and time spent to reach the target serve as primary indices of cognitive performance.

The overall objectives of the current research were to develop the VAST as a novel rat task that (a) measures spatial navigation performance while averting confounds typically associated with appetitive or aversive motivational cues; (b) is straightforward, based on intrinsic behavior, and quickly learned; (c) manifests performance outcomes that are substantially degraded by total SD; and (d) produces large effects so that relatively small sample sizes are required to detect performance changes. To address these goals, we performed three separate experiments. In experiment 1, we used a place preference paradigm to measure whether rats exhibit greater preference for vibration over other widely used adverse stimuli: water, bright light, or foot shock. In experiment 2, we exposed rats to vibration, water, bright light, foot shock, or no stimulus (control), and then measured the concentration of circulating corticosterone as a marker of physiological stress. In experiment 3, we determined whether the VAST can be learned quickly by administering it to rats over three consecutive days. On the third day, half of the rats were sleep deprived for 6 h to determine whether VAST performance is sensitive to SD-induced performance impairments.

## 2. Experiment 1: Place preference

### 2.1. Methods

#### 2.1.1. Animals

Breeder rat pairs were procured from Envigo (South Kent, WA) and colonized locally. Twelve male Long Evans rats (three cohorts of four; aged 8−12 weeks) were used in experiment 1. Each cohort was randomized to one of three place-preference conditions: vibration vs. water (n = 4), vibration vs. foot shock (n = 4), and vibration vs. bright light (n = 4). The rats were housed two to a cage (i.e., two cages for each cohort), with access to food and water *ad libitum*. The cages were kept in a colony room with a 12:12 h light:dark cycle and an average daily temperature of 24 ± 2°C. All animal procedures were approved by Washington State University’s Institutional Animal Care and Use Committee (IACUC) and were compliant with National Institutes of Health (NIH) guidelines.

#### 2.1.2. Apparatus and equipment

**Fig 1:**
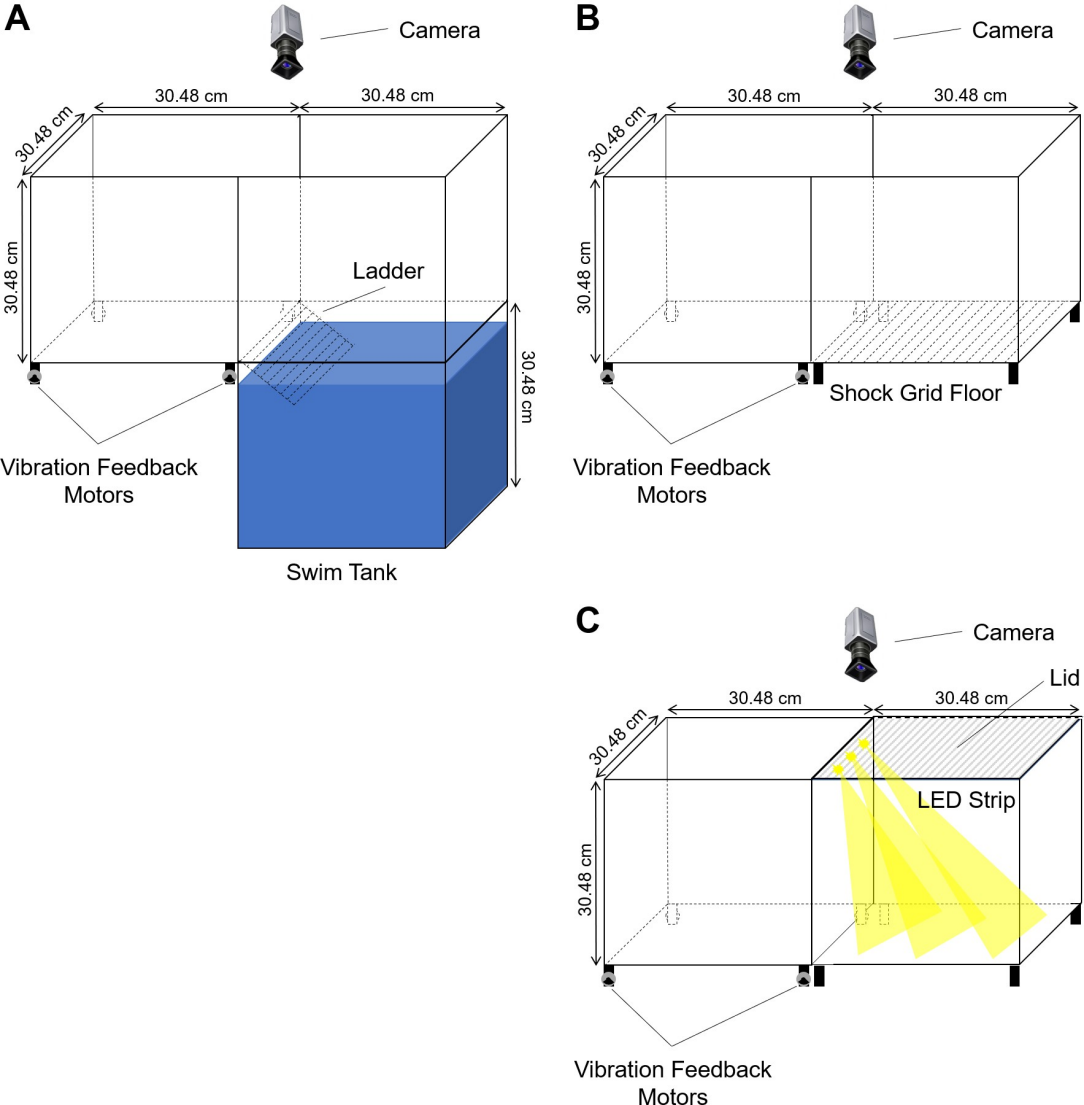
Schematics of the apparatuses used in experiments 1 and 2. Schematics of the apparatuses used to compare (A) vibration vs. water, (B) vibration vs. shock, and (C) vibration vs. light in experiment 1 are shown. Comparable box components were used for experiment 2, but the boxes were enclosed and used without an adjacent box to subject the rat to each stimulus independently.

Experiment 1 was performed in 30.48 x 60.96 x 30.48 cm polycarbonate containers with a vibration side and an alternative side with water, bright light, or foot shock (Fig 1). The dimensions of these containers were selected so that environments would be equivalent in size to the rod grid shock floor (Coulbourn Instruments H13-16 (REV A) Precision Regulated Animal Shocker). The containers were painted tan to provide enough contrast to distinguish the rat’s position accurately, and an overhead camera recorded a video of the rat’s movements, which were analyzed by ANY-maze v.6.16. The vibration side of the container had four weight offset motors (model 334–401; Precision Microdrives) attached to the bottom, which produced floor vibrations. The motors reached approximately 2,700 RPM, as measured by a tachometer. The force of the vibration was 1.2 *g* on average, with a maximum of 1.8 *g*, as measured with an accelerometer affixed to the base of the platform. FFT analyses of the output demonstrated a frequency range of 0–3.24 Hz. The auditory output from the vibration motors was 63 ± 2 dB.

For the alternative side of the container in the water version, the box was filled with water (Fig 1A). The water temperature was held at 25°C ± 1°C to avoid any undue stress caused by thermoregulation challenges. The surface was approximately 1 cm below the adjacent vibration floor, with a ladder (15.2 cm x 30.5 cm) placed in the water container adjacent to the vibration floor. For the alternative side of the container in the foot shock version, there was a rod grid shock floor (Coulbourn Instruments H13-16 (REV A) Precision Regulated Animal Shocker) (Fig 1B). This floor produced 0.5 mA shocks for 0.5 s at 5 s intervals. For the alternative side of the container in the bright light version, LED strips were mounted to the top of the box and aimed downward away from the vibration side, and there was a lid to help disperse the light evenly (Fig 1C). The LED strips provided 900 ± 84 lux illumination within the box.

#### 2.1.3. Pre-experimental protocol

Before each habituation or experimental session, the rats’ home cages were moved to the experiment room, and the apparatus and working area were cleaned with 10% ethanol and Spor-Klenz (Steris Life Sciences), respectively. The entire apparatus was cleaned with 10% ethanol between trials to remove any residual olfactory cues.

#### 2.1.4. Handling and habituation

Handling and habituation sessions began at Zeitgeber time (ZT) 10, which is 10 hours after light onset. Rats were handled for 5 min a day on five consecutive days, beginning seven days before experimentation. On the fourth and fifth days, the rats were handled, and then habituated to one side of their respective apparatus (depending on condition) in the absence of stimuli (i.e., no vibration, water, shock, or lights) for 5 min. Rats were habituated to one side of the apparatus (vibration or alternative) on each habituation day, with the order determined randomly.

#### 2.1.5. Experimental design

The experimental session began at ZT 10. Each rat completed two place-preference trials. For each trial, the rat was placed facing the outside wall of one side of the apparatus, then left to explore for 120 s. After 120 s, the rat was removed from the arena and placed back in its home cage. If a rat was wet from the water condition, it was dried with a towel before being returned to its home cage. After all rats completed their first trial, each rat completed the second trial (in the same subject order) with the starting position on the opposite side of their first trial. Thus, data from two trials with opposite starting positions, in counterbalanced, randomized order, were collected for each rat. All rats were returned to the colony room following experimentation.

#### 2.1.6. Measurements

The percent of time that the rats spent on the vibration side of the arena was measured to assess overall place preference. The number of entrances to each side (i.e., the number of times they crossed between sides of the container) and the average dwell duration (i.e., the average time spent on each side before entering the other side) were recorded to assess exploratory behavior. For the vibration vs. light condition, the lid obscured the camera’s view on the light side, but the number of entrances and dwell duration could be inferred from the observations on the vibration side.

**Fig 2:**
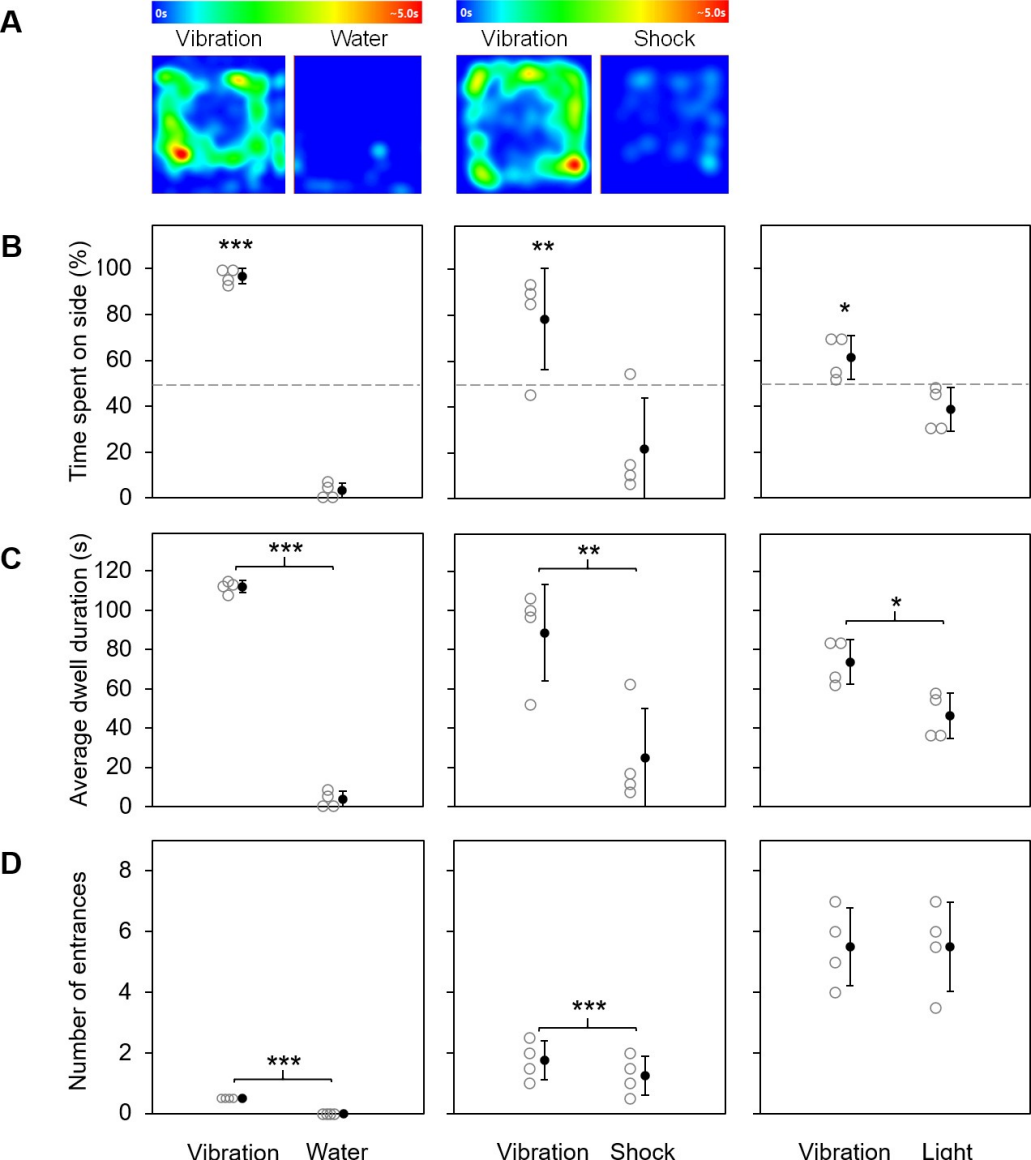
Rats prefer vibration over other stimuli. Results from experiment 1 (place preference) are shown. (A) Heat maps showing average dwell time for the vibration vs. water (left) and vibration vs. shock (middle) conditions. (B-D) Individual data points (open gray markers) and group means ± SE (solid black markers) for (B) percent time spent on each side (significance is indicated for vibration side relative to 50%), (C) average dwell duration per entrance on each side, and (D) number of entrances to each side (SEs are zero in the vibration vs. water comparison). *p<0.05, **p<0.01, ***p<0.001.

A dwell-time heat map was constructed in ANY-maze for the vibration vs. water and vibration vs. foot shock conditions as a visual representation of the rat’s exploration of the place preference apparatuses (Fig 2A). Since the lid on the light side obscured the infrared camera images in the light condition, no dwell-time heat map could be made for the vibration vs. light condition.

#### 2.1.7. Statistical analysis

Data for each rat was averaged over trials. Then, as the primary analysis, place preference for the vibration side over the alternative side was investigated for each condition (i.e., vibration vs. water, vibration vs. shock, and vibration vs. light). A one-sided, one-sample *t*-test was used to determine whether the total percent of time spent on the vibration side exceeded 50%. As secondary analyses, the number of entrances and the dwell duration per entrance were investigated. One-sided, paired-sample *t*-tests were used to determine whether the number of entrances and dwell duration for the vibration side exceeded those for the alternative sides. Effect sizes were calculated using Cohen’s *d* [11].

One rat in the vibration vs. shock condition did not explore the apparatus during one of the trials. Both of this rat’s sessions were therefore excluded, leaving n = 3 (i.e., 6 trials total) for the analysis of the vibration vs. shock condition.

### 2.2. Results

Rats spent significantly more than 50% of their time on the vibration side of the apparatus in the vibration vs. water condition (t_3_ = 27.72, *p*<0.001), vibration vs. foot shock condition (t_2_ = 16.26, *p* = 0.002), and vibration vs. light condition (t_3_ = 2.39, *p* = 0.048) (Fig 2B). Additionally, the average dwell duration per entrance was significantly greater for the vibration side in the vibration vs. water (t_3_ = 32.29, *p*<0.001, *d* = 1.37), vibration vs. shock (t_2_ = 16.19, *p* = 0.002, *d* = 1.32), and vibration vs. light (t_3_ = 2.39, *p* = 0.048, *d* = 0.44) conditions (Fig 2C). Finally, there were no entrances to the water side in the vibration vs. water condition (*d* = 1.41); and for every rat, the number of entrances was exactly 1 greater in the vibration condition than in the shock condition (*d* = 0.28). Entrances did not differ for the vibration vs. light condition (t_3_ = 0.00, *p* = 0.50, *d* = 0.00) (Fig 2D). Taken together, these results indicate that rats exhibited place preference for vibration over water, foot shock, and bright light environments (S1 Table).

## 3. Experiment 2: Corticosterone response

### 3.1. Methods

#### 3.1.1. Animals

Breeder rat pairs were procured from Envigo (South Kent, WA) and colonized locally. Forty-two male Long Evans rats (eighteen cohorts of two or three; aged 8−12 weeks) were used in experiment 2. Each cohort was randomized to one of five exposure conditions: vibration (3 cohorts; n = 9), water (3 cohorts; n = 9), light (4 cohorts; n = 8), shock (4 cohorts; n = 8), and no-stimulus control (4 cohorts; n = 8). Each cohort was housed within one cage and had the same housing conditions as described for experiment 1 (see section 2.1.1).

#### 3.1.2. Apparatus and equipment

A divider was placed between each side of the containers used in experiment 1 (Fig 1), such that there were isolated open fields with vibration, water, foot shock, and light. The containers were identical to those used in experiment 1. However, for the water box, the water level was 5 cm lower than in experiment 1 and the ladder was removed so that the rats remained in the water for the entire duration of the trial. For the light box, the LEDs were mounted on the center of the lid and the light was directed downward to provide even platform coverage.

#### 3.1.3. Pre-experimental protocol

The pre-experimental procedures were identical to those used in experiment 1 (see section 2.1.3).

#### 3.1.4. Handling and habituation

Handling and habituation sessions began at ZT 8 and were identical to those used in experiment 1 (see section 2.1.4) except that the rats were exposed to the same apparatus on both habituation days.

#### 3.1.5. Experimental design

During the experimental session, which occurred at ZT 8, each rat was placed in the box corresponding to its assigned condition for 120 s in the presence of the stimulus (i.e., vibration, water, foot shock, or bright light). The rat was then removed from the box and placed in a transport cage containing bedding from its home cage (to avoid any stress associated with novel bedding), then it was immediately carried to a procedure room where trunk blood was collected by live decapitation. The same procedure was used for the no-stimulus control condition except that rats in this condition were not placed in an apparatus before trunk blood collection.

#### 3.1.6. Measurements

Blood was collected in EDTA vials. Each vial was immediately inverted 20 times, then centrifuged at 2,200−2,500 RPM for 15 min while at a temperature of 4°C. Once centrifuged, each supernatant was transferred into a fresh 1.5 ml snap-cap tube and the plasma was frozen at -80°C until corticosterone assessment. Corticosterone levels were quantified by ELISA (Enzo, ADI-900-097), following the manufacturer’s instructions. The samples were run on two 96-well plates, with appropriate standards and controls (following standard procedures; Enzo, ADI-900-097). Duplicate samples from the bright light and foot shock conditions were placed on plate 1, and duplicate samples from the water and no-stimulus control conditions were placed on plate 2. Duplicate samples from the vibration condition were placed on both plates to account for any between-plate differences in the measurements of corticosterone concentrations.

For each well, the bound optical density for each sample and standard was measured at 405 nm and the noise was averaged over measurements at 570 and 590 nm. The average noise was subtracted from the bound optical density of each well, and the adjusted average bound optical density was used to determine the percent corticosterone bound (using standard procedures; Enzo, ADI-900-097). The corticosterone concentration for each sample was calculated using a standard curve plotted from the known corticosterone concentrations of 5 standards on each plate. For data analysis, corticosterone concentration was averaged across wells for each subject on each plate.

#### 3.1.7. Statistical analysis

Differences in corticosterone concentrations were first assessed using one-way analysis of variance (ANOVA) as a function of condition, controlling for plate. A planned contrast was used to verify that for the vibration condition, corticosterone concentration did not differ significantly between the two plates. Planned contrasts were then performed between the vibration condition and each of the other conditions (i.e., between-groups) to determine whether vibration was more or less physiologically stressful than the other stimuli. Effect sizes were calculated using Cohen’s *d* [11].

One rat in the water condition was excluded because it was swimming below the surface with its head submerged for a large portion of the trial; thus, n = 8 rats were included in the analysis of the water condition.

### 3.2. Results

**Fig 3:**
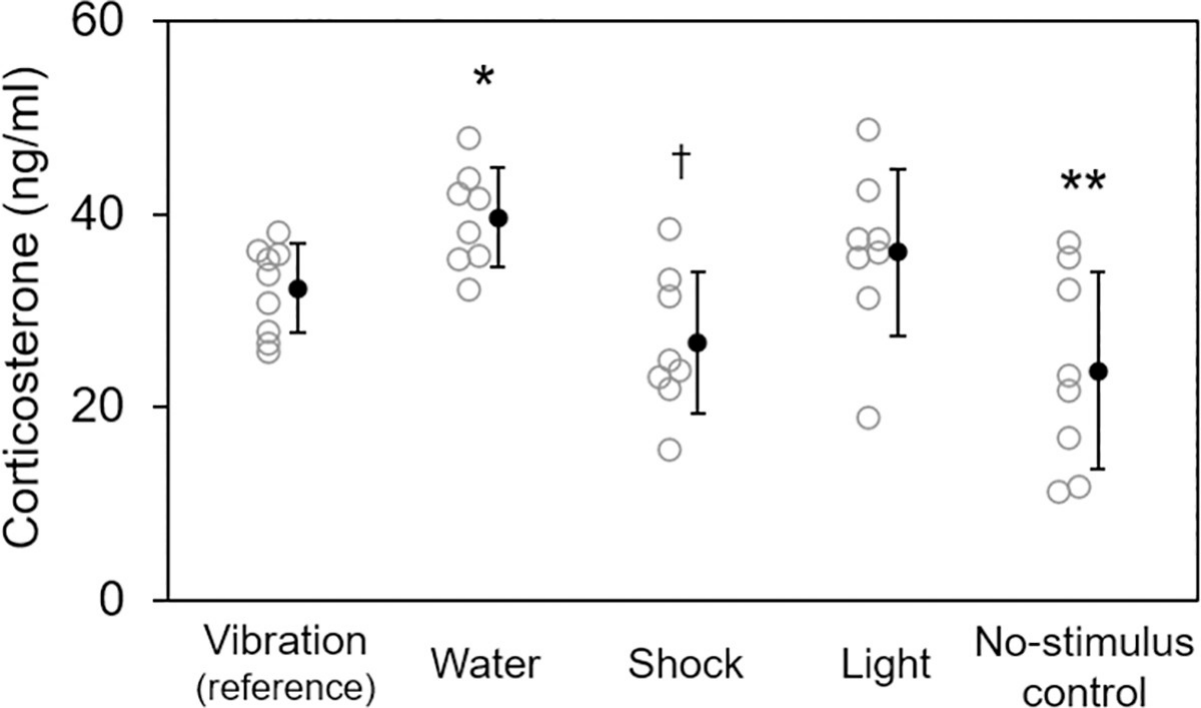
Corticosterone levels following exposure to different stimuli compared to vibration. Results from experiment 2 (corticosterone response) are shown as individual data points (open gray markers) and group means ± SE (solid black markers). Statistical significance is indicated above each condition for the comparison to vibration (reference) by planned contrast. ^†^*p*<0.1, *p<0.05. **p<0.01.

There was a significant effect of condition on the corticosterone concentrations observed in this experiment (F_4,44_ = 6.93, *p*<0.001) (Fig 3). As expected, there were no significant differences in the corticosterone concentrations measured between plates for the vibration condition (F_1,44_ = 1.15, *p* = 0.29). Compared to the vibration condition, the average corticosterone concentration was significantly higher for rats subjected to the water condition (F_1,44_ = 6.00, *p* = 0.018, *d* = 0.20). In contrast, there was a trend for the corticosterone concentration to be lower in rats in the foot shock condition compared to the vibration condition (F_1,44_ = 3.66, *p* = 0.062, *d* = 0.19). No significant difference was observed between the corticosteroid concentrations measured in the bright light and vibration conditions (F_1,44_ = 1.53, *p* = 0.22, *d* = 0.11). Finally, the vibration condition exhibited a greater average corticosterone concentration than the no-stimulus control condition (F_1,44_ = 8.21, *p* = 0.006, *d* = 0.30) (S2 Table).

The finding of a trend toward lower physiological stress induced by foot shock versus vibration, as measured by corticosterone, was unexpected and may reflect the relatively low foot shock intensity and frequency employed here. Increasing the foot shock intensity to 1−2 mA or the frequency to 0.5−1 Hz, which would still be within accepted limits in the fields of neuroscience and psychology, would likely cause a greater increase in circulating corticosterone. This consideration notwithstanding, the results of experiment 2 indicate that floor vibration was more physiologically stressful than exposure to no stimuli, but less stressful or at least no more stressful than other motivational stimuli commonly used in rat performance task paradigms.

## 4. Experiment 3: VAST performance

### 4.1. Methods

#### 4.1.1. Animals

Breeder rat pairs were procured from Envigo (South Kent, WA) and colonized locally. Fifteen male Long Evans rats (four cohorts of three or four; aged 8−12 weeks), with the same housing conditions as described for the first experiment (see section 2.1.1), were used in experiment 3. In this experiment, rats performed the VAST—an open-field task in which gradient floor vibration provides motivation for the rodent to navigate in the direction of diminishing vibration to where an unmarked target destination is located. Each cohort was randomized to either sleep deprivation (SD; n = 8) or control (CONT; n = 7).

#### 4.1.2. Apparatus and equipment

**Fig 4:**
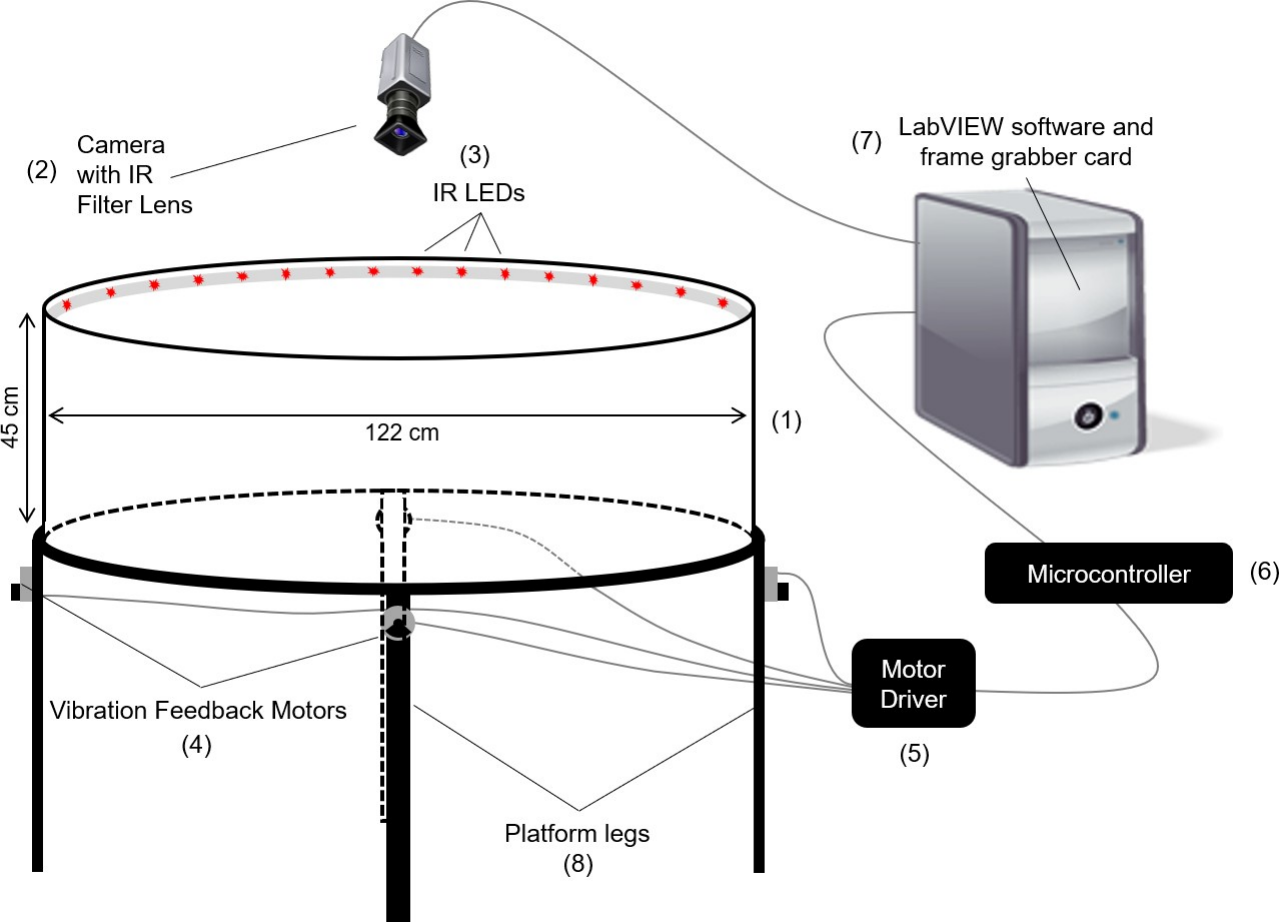
Schematic of the VAST apparatus used in experiment 3. The VAST apparatus used in experiment 3 is shown, including the following primary components: (1) walled, circular, open field arena, (2) Manta G-201 digital camera with infrared filter (Allied Vision), (3) SMD5050-300-IR infrared Tri-Chip flexible LED strip (LEDLightsWorld) surrounding the maze rim, (4) four 45 mm electric rotary motors (model 345–400; Precision Microdrives), (5) two TB6612FNG dual motor driver carriers (Pololu), (6) chipKIT WF32 microcontroller (Digilent), and (7) computer with LabVIEW 2012 software and PCIe-6341 multifunction I/O card (National Instruments).

The VAST was executed in a black polycarbonate circular arena (122 cm diameter) with white acrylic walls (45 cm high) mounted on four support legs (Fig 4). The arena was raised so that visual cues from an experimenter or location of the computer, etc., could not be used as extramaze cues. Four 45 mm weight-offset rotary motors, each attached to the base of one of the four support legs, provided the vibratory stimulus to the VAST arena. Additionally, the vibration from the motors provided an auditory stimulus, which was 70 ± 2 dB.

**Fig 5:**
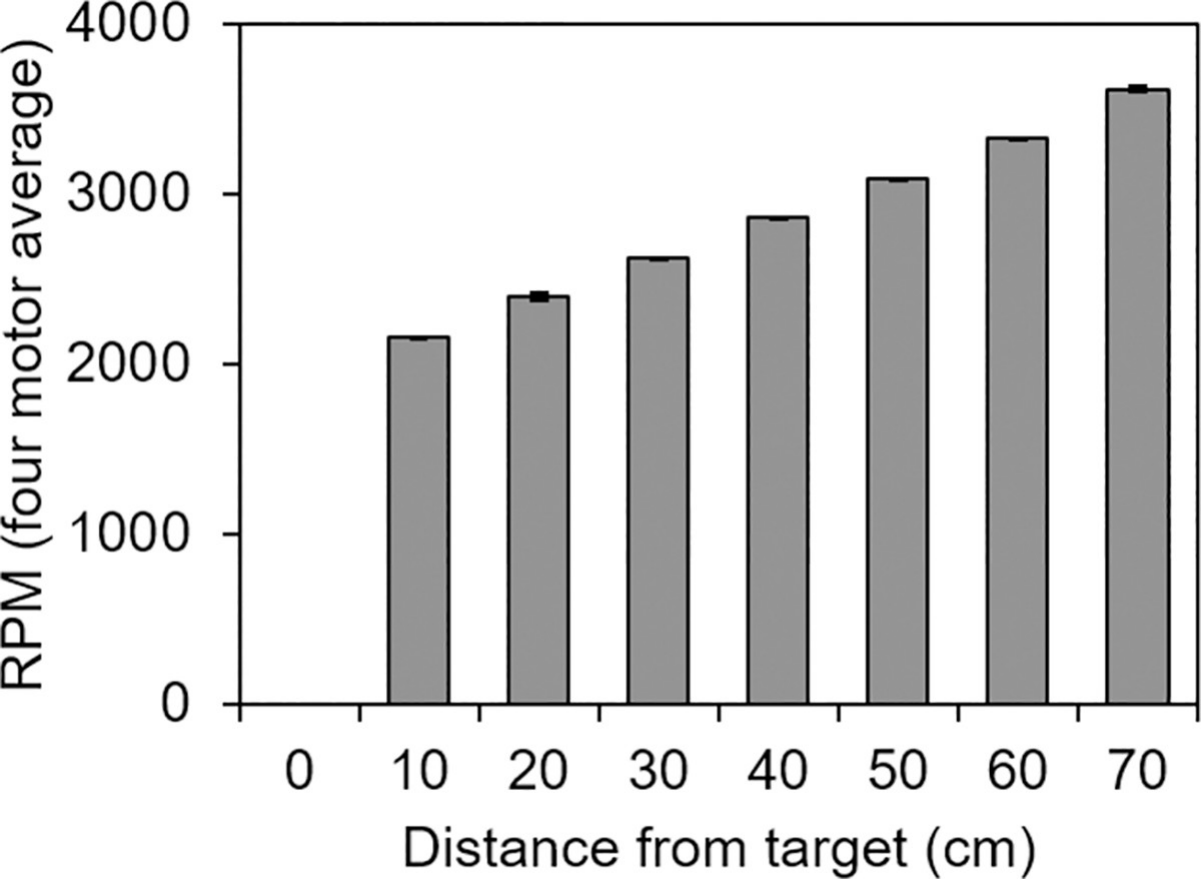
Vibration gradient for experiment 3. VAST vibration motor frequencies, averaged over the four vibration motors, are shown as a function of distance from the target. Motor frequencies (in revolutions per min) were measured with a tachometer (sampled in triplicate for each motor) while a stationary object was moved away, in 10 cm increments, from the outer edge of a randomly selected target destination.

A pulse width modulator governed the DC current output that controlled the duty cycle of each motor via the LINX vi package (LabVIEW, MakerHub). A software subroutine implemented in LabVIEW calculated the distance between the centroid position of the rat, determined as described below, and the unmarked target destination, and then used this information to control the frequency of the motors (range: 0−3,500 RPM). Slower vibrations indicated less distance to the target. Fig 5 shows the relationship between distance to the target and the motor frequency.

VAST experiments were performed in dim red light, which was provided by the room (25−30 lux) and the VAST apparatus itself (supplied by a downward-angled strip of infrared LEDs mounted around the top of the arena’s wall) (Fig 4). These lights provided comprehensive platform coverage that was sufficient for motion tracking and obscured extramaze visual cues from the rats. Each trial was video recorded digitally through an infrared filter to determine the rat’s position, drive the vibration motors, and capture data for analysis of trial performance. A Manta Vision camera was used to acquire a video feed of each trial at 10 frames per second. Image acquisition was implemented in LabVIEW using the NI-IMAQdx 4.0 driver software (National Instruments). The video feed was filtered based on a predetermined pixel intensity threshold to distinguish the rat from the platform. From the resulting image, a center of mass algorithm was used to define a centroid, which was then used as an estimate of the rat’s position. A criterion of at least 1-pixel (approximately 1 mm) change in position was used to determine whether the rat was moving between frames [12].

Olfactory cues were minimized by cleaning the maze with 10% ethanol between trials. Assignments of entry points and target destinations for each trial were randomized. These measures were taken to restrict cue modality to the tactile and auditory stimuli of the vibrations.

#### 4.1.3. Pre-experimental protocol

The pre-experimental procedures were identical to those used in experiment 1 (see section 2.1.3).

#### 4.1.4. Handling and habituation

Handling and habituation sessions began at ZT 6 and were identical to those used in experiment 1 (see section 2.1.4), except that rats were exposed to the VAST apparatus—placed in the open field in the absence of vibration—on both habituation days.

#### 4.1.5. Experimental design

Each rat performed 9 trials of the VAST on each of the three experiment days, for a total of 27 trials over the three days. Rats were left undisturbed before VAST testing on all experiment days, except that rats in the SD group were sleep-deprived for 6 h by gentle handling in their home cage beginning at ZT 0 (light onset), immediately before testing on day 3.

**Fig 6:**
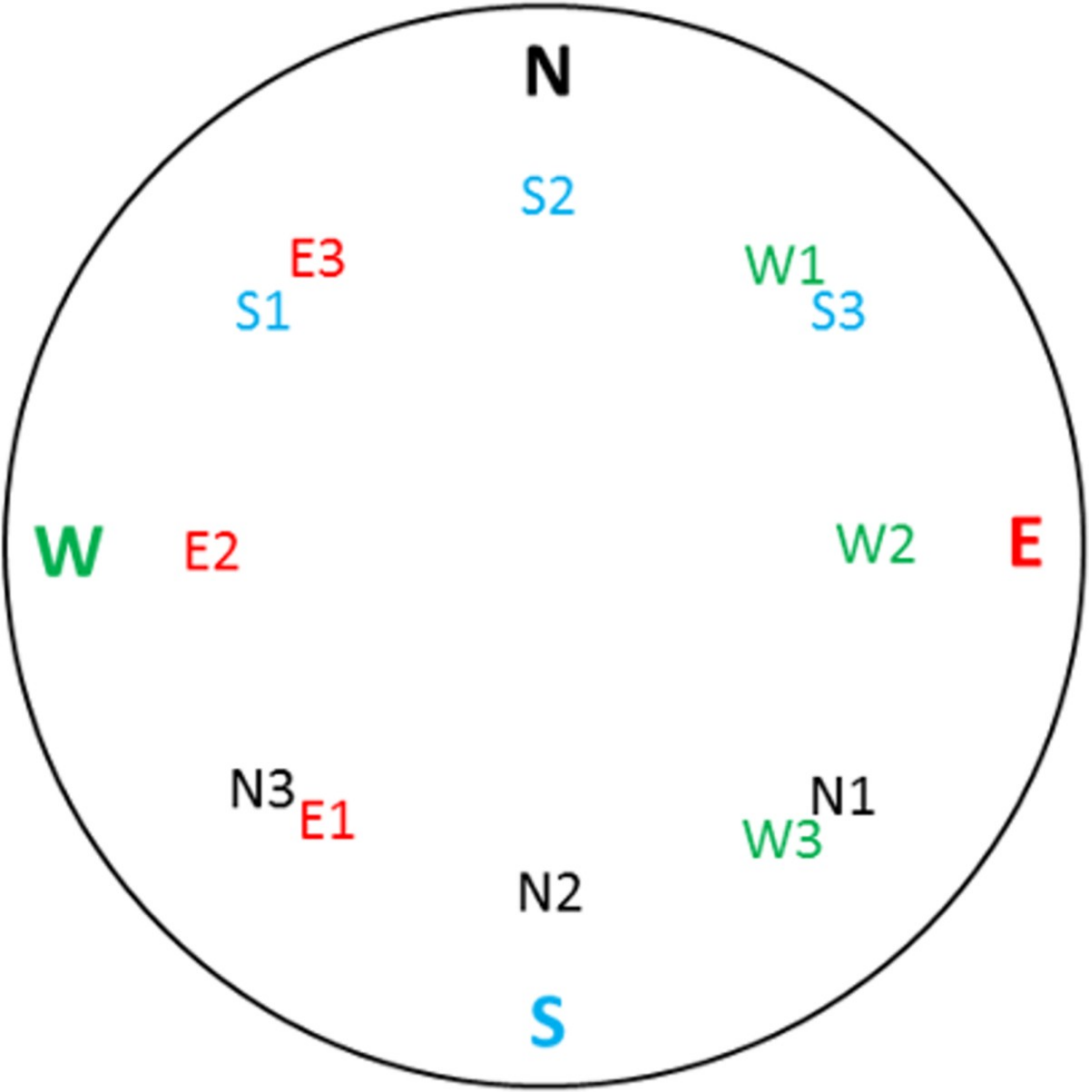
Insertion/target map for experiment 3. VAST arena insertion points (N = North, W = West, E = East, and S = South) are shown with associated, equidistant target destinations (N1, N2, N3 for entry point N, W1, W2, W3 for entry point W, etc.).

At the beginning of each trial, the rat was removed from the home cage and gently placed facing the outside wall at one of four entry points that were labeled North, East, South, and West (Fig 6). The experimenter’s hand was immediately withdrawn, and vibrations and trial tracking began as soon as the hand was out of the video image. For the duration of the trial, the experimenter remained silent and did not move. There were three target destinations per entry point (depicted as N1, N2, N3, E1, etc.) (Fig 6). Each target destination was 25 cm in diameter (area ≈ 491 cm^2^) and equidistant from its corresponding entry point. Target destination borders were at least 15 cm from the wall; thus, the target was located far enough away from the wall to prevent discovery via thigmotaxis. For every day of performance testing, entry points and target destinations were quasi-randomly selected using a Latin square so that the gradient floor vibrations were the only cue to locate the target destination, and memory of prior search strategies would not aid in finding the target location.

The vibrations ceased and the trial ended when a part of the rat’s body (excluding the tail) entered the target destination (the rat was not required to stop within the target destination), or when 120 s of searching passed without the rat locating the target (indicating that the rat did not use the gradient vibration cues to locate the target). After the vibrations ceased, the rat was returned to its home cage.

Rats alternated trials with their cage mates (two or three rats per cage, depending on the cohort). For cohorts with four rats, the two rats in one cage completed all nine trials, then the second pair began trials immediately. The average inter-trial interval for each rat during each session was 3.61 ± 0.78 minutes. The order of trial initiation for each group was the same for all three days, and no more than four rats were run on a single day. After each rat completed all nine trials for the session, two Froot Loops per rat were placed in each cage and the rats were returned to the colony room.

#### 4.1.6. Statistical analyses

For experiment 3, the primary dependent variables measuring VAST performance were success rate, path distance (i.e., the distance covered in search of the target), and time to target. Area under the curve of path distance by time to target (AUC; m∙s), time still, percent of time still, percent of time moving toward the target, and speed were analyzed as secondary variables. Success rate was calculated as the percent of trials in which the rat located the target before the end of the 120 s trial. Path distance (m) and trial time (s) were reported every 0.1 s by the LabVIEW software. As a way to capture performance improvements in path distance, trial time, or both, within a single dependent variable, the area under the curve (AUC) was calculated using the cumulative distance traveled every 0.1 s during the trial.

The first 0.3 s of each trial were excluded from the AUC analysis because the start-of-trial tracking calibrations resulted in less accurate position estimates. Additionally, two motion tracking errors occurred in one trial, so the distances traveled at those times were replaced with the average distance traveled per 0.1 s from the four nearest valid position estimates.

Time still was defined as the total amount of time the rat spent immobile or moving an inconsequential amount (e.g., a position change merely due to a non-ambulatory head movement). It was assessed as any 0.1 s interval with a change in position of less than 15 pixels (approximately 1.5 cm). The percent of time moving toward the target was calculated as the percent of time moving toward the target out of the total time moving (time moving toward or away from the target), and the percent of time still was calculated as the percent of time still out of the total time to reach the target. Time moving toward the target was assessed as the total amount of time that the rat’s distance from the target decreased by more than 15 pixels in 0.1 s. Time moving away from the target was assessed as the total amount of time that the rat’s distance from the target increased by more than 15 pixels in 0.1 s. Note that time moving toward the target and time moving away from the target were not analyzed independently and were only used for the calculation of the percent of time moving toward the target. Speed was assessed as path distance divided by time to reach the target.

For experiment 3, one rat was excluded from the data analysis because its success rate was extremely low on day 2 (below 75%), indicating inadequate task retention. Thus, fourteen rats were included in the data analysis (SD: n = 7; CONT: n = 7).

For the primary data analysis, trial data were averaged for each rat by day. Data were analyzed using mixed-effects ANOVA with fixed effects of day and group, with cohort as a covariate, and a random effect of subject on the intercept. To assess for changes in performance of well-rested rats over days planned contrasts were performed between day 1 and 2 for all rats, and between day 2 and 3 for the CONT group. Between-groups planned contrasts were used to measure the impact of SD versus CONT on day 3. All rats were analyzed as a single group for comparisons between days 1 and 2, whereas SD and CONT groups were differentiated for between-group comparisons on day 3. Effect sizes of between-groups comparisons on day 3 were calculated using Cohen’s *d* [11].

Secondary analysis was used to determine whether performance changed within-day on days 1 and 2. Average path distance, time to target, and AUC for groups of three consecutive trials were calculated for each rat such that there were three trial blocks for each day of VAST performance. The trial block data was analyzed by mixed-effects ANOVA with fixed effects of day, trial block, and cohort, and a random effect of subject on the intercept. Planned contrasts between trial blocks 1 and 2, trial blocks 2 and 3, and trial blocks 1 and 3 assessed within-day changes in performance on days 1 and 2 (when all rats were well-rested).

### 4.2. Results

#### 4.2.1. VAST results overall

**Fig 7:**
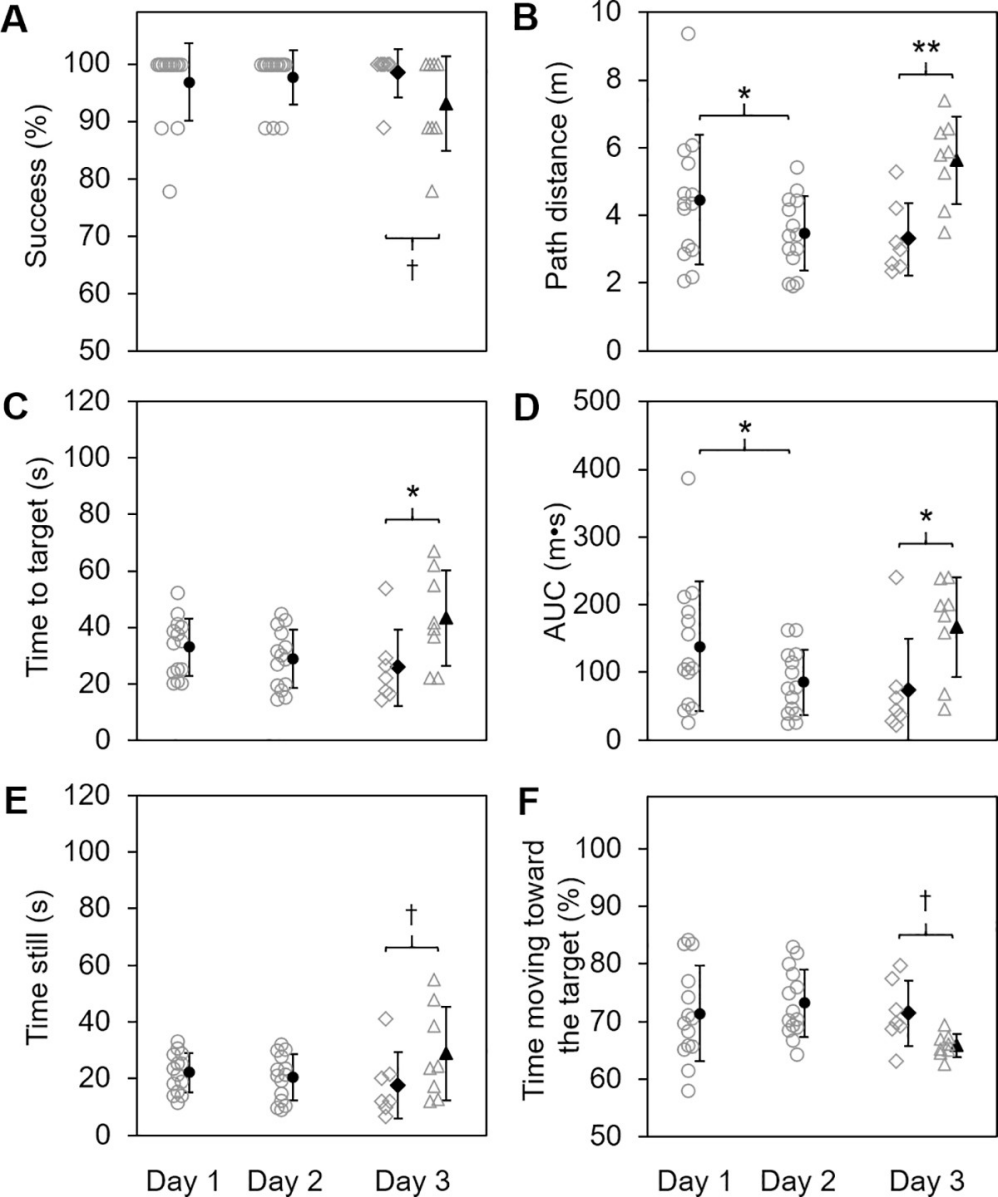
Rapid learning of the VAST and impairment of VAST performance by SD. Results from experiment 3 (VAST performance) are shown. Data are plotted as individual data points (open gray markers) and group means ± SE (solid black markers) for all rats (SD and CONT groups) combined on days 1 and 2 (circular markers) as well as for CONT (diamond markers) and SD (triangle markers) groups separately on day 3. Statistically significant differences between days 1 and 2 (all rats) are indicated by an asterisk. Statistically significant differences between SD and CONT groups on day 3 are indicated by a cross or an asterisk. ^†^*p*<0.1, *p<0.05, **p<0.01.

Group results for VAST performance are shown in Table 1. Rats immediately located the target on nearly all VAST trials, as evidenced by success rates exceeding 95% on days 1 and 2 (Fig 7A). For the primary variables, mixed-effects ANOVA of success rates revealed a trend for the main effect of group (F_1,20_ = 3.67, *p* = 0.070), but no significance for the main effect of day (F_2,20_ = 0.40, *p* = 0.68) or the interaction of day and group (F_5,20_ = 1.20, *p* = 0.34). Path distance was significantly affected by day (F_2,20_ = 4.29, *p* = 0.028) and group (F_1,20_ = 7.80, *p* = 0.011), and the interaction of day and group was also significant (F_5,20_ = 4.41, *p* = 0.007) (Fig 7B). Time to target was significantly affected by group (F_1,20_ = 7.79, *p* = 0.011) and there was a trend for an interaction of day and group (F_5,20_ = 2.21, *p* = 0.093), but the effect of day was not significant (F_2,20_ = 0.78, *p* = 0.47) (Fig 7C) (S3A Table).

**Table 1:** VAST performance results from experiment 3.

	Day 1, all	Day 2, all	Day 3, CONT	Day 3, SD
Dependent variables	N = 14	N = 14	n = 7	n = 7
Success (%)	96.83 (1.82)	96.83 (1.39)	98.41 (1.59)	92.06 (3.17)
Path distance (m)	4.46 (0.51)	3.47 (0.29)	3.30 (0.41)	5.59 (0.52)
Time to target (s)	32.96 (2.73)	28.90 (2.78)	25.61 (5.14)	43.76 (6.86)
AUC (m·s)	138.19 (25.60)	85.23 (12.80)	73.09 (28.97)	168.10 (29.84)
Time still (s)	22.20 (1.84)	20.47 (2.16)	17.65 (4.40)	29.53 (6.63)
Time still (%)	60.83 (2.07)	62.38 (2.59)	61.56 (5.23)	60.30 (5.16)
Time moving toward the target (%)	71.38 (2.21)	73.24 (1.57)	71.46 (2.13)	65.99 (0.82)
Speed (m/s)	0.15 (0.01)	0.15 (0.01)	0.16 (0.02)	0.15 (0.02)

VAST performance for all rats on days 1 and 2, and the CONT and SD groups separately on day 3, are shown. Data are reported as means (SE).

For the secondary variables, the AUC also showed an effect of group (F_1,20_ = 8.60, *p* = 0.008) along with a significant interaction of day and group (F_5,20_ = 2.96, *p* = 0.037), but the effect of day was not significant (F_2,20_ = 2.36, *p* = 0.12) (Fig 7D). For time still, there was a trend for the main effect of group (F_1,20_ = 3.89, *p* = 0.063), but the effect of day (F_2,20_ = 0.25, *p* = 0.78) and the interaction of day and group (F_5,20_ = 1.15, *p* = 0.37) were not significant (Fig 7E). We also assessed time still as a percent of the total time spent in the apparatus and found that it did not show any significant effects of day (F_2,20_ = 0.10, *p* = 0.90) or group (F_1,20_ = 0.15, *p* = 0.70) or the interaction of day and group (F_5,20_ = 0.11, *p* = 0.99). Percent of time moving toward the target did not show any significant effects of day (F_2,20_ = 2.49, *p* = 0.11) or group (F_1,20_ = 2.86, *p* = 0.11) or any interaction of day and group (F_5,20_ = 2.12, *p* = 0.11) (Fig 7F). Speed also did not show any significant effects of day (F_2,20_ = 0.14, *p* = 0.87) or group (F_1,20_ = 0.00, *p* = 0.97) or the interaction of day and group (F_5,20_ = 0.26, *p* = 0.93) (S3B Table).

Overall, rats averaged 4.8 min of vibration exposure per day. Rats that had more than two failed trials after SD still experienced vibration for 10 min or less in a single day.

#### 4.2.2. VAST learning and retention over days

For the primary variables, success rate was stable from day 1 to day 2 (Fig 7A). Path distance decreased by approximately 1 m for all rats from day 1 to day 2 (F_1,20_ = 7.18, *p* = 0.014) and did not change from day 2 to day 3 for the CONT group (Fig 7B). Time to target was stable from day 1 to day 2 for all rats and remained stable from day 2 to day 3 for the CONT group (Fig 7C).

For the secondary variables, the AUC decreased from day 1 to day 2 for all rats by about 50 m∙s (F_1,20_ = 4.72, *p* = 0.042) and was stable from day 2 to day 3 for the CONT group (Fig 7D). Time still (Fig 7E), percent of time still, percent of time moving towards the target (Fig 7F), and speed were stable over days 1 and 2 for all rats and from day 2 to day 3 for the CONT group.

Improvements in performance over days 1 and 2 suggest that the subjects learned to find the unmarked target location more effectively over days, and the stability of performance over days 2 and 3 in the CONT group indicate maximal performance on day 2 and the retention of VAST learning over consecutive days of testing.

#### 4.2.3. VAST learning over trial blocks

Analysis of performance over trial blocks for days 1 and 2 revealed that there was a trend for decreased path distance from trial block 1 to 2 (F_1,80_ = 2.82, *p* = 0.097) and a statistically significant decrease from trial block 2 to 3 (F_1,80_ = 5.60, *p* = 0.020). Similarly, the time to target showed a trend toward a decrease from block 1 to 3 (F_1,80_ = 3.54, *p* = 0.063), but the incremental changes from block 1 to 2 and block 2 to 3 were not significant. The decrease in AUC from block 1 to 2 approached statistical significance (F_1,80_ = 2.87, *p* = 0.094), with no change from block 2 to 3; however, there was a significant improvement overall from block 1 to 3 (F_1,80_ = 10.97, *p* = 0.001). Performance over trial blocks was stable for path distance, time to target, and AUC on day 2, indicating that there was no within-session learning after day 1. These results show that rats performed the VAST increasingly more efficiently over the course of day 1 and maintained that performance throughout day 2, indicating that improvements within-session occurred only on the first day of VAST performance testing.

#### 4.2.4. SD effects on VAST performance

For the primary variables on day 3, the SD group had a lower success rate compared to the CONT group, but this between-groups comparison did not reach statistical significance (F_1,20_ = 3.29, *d* = 0.07, *p* = 0.085) (Fig 7A). Path distance was also found to be significantly longer in the SD group versus the CONT group (F_1,20_ = 9.39, *d* = 0.50, *p* = 0.006) (Fig 7B), and the SD group searched for the target about 20 s longer than the CONT group (F_1,20_ = 7.30, *d* = 0.51, *p* = 0.014) (Fig 7C).

For the secondary variables, the AUC for the SD group was approximately 100 m∙s greater than for the CONT group (F_1,20_ = 5.82, *d* = 0.73, *p* = 0.026) (Fig 7D). In addition, the SD group remained still approximately 10 s longer than the CONT group, though this comparison did not reach statistical significance (F_1,20_ = 4.26, *d* = 0.49, *p* = 0.052) (Fig 7E). For the percent of time still, there was no difference between groups on day 3. There was a trend for decreased percent time moving toward the target for the SD group compared to the CONT group (F_1,20_ = 3.01, *d* = 0.08; *p* = 0.098) (Fig 7F). Finally, there was also no difference in speed between groups. These analyses of between-groups differences on day 3 revealed that 6 h of SD causes substantial and robust performance decrements on the primary VAST outcomes despite relatively small sample sizes.

**Fig 8:**
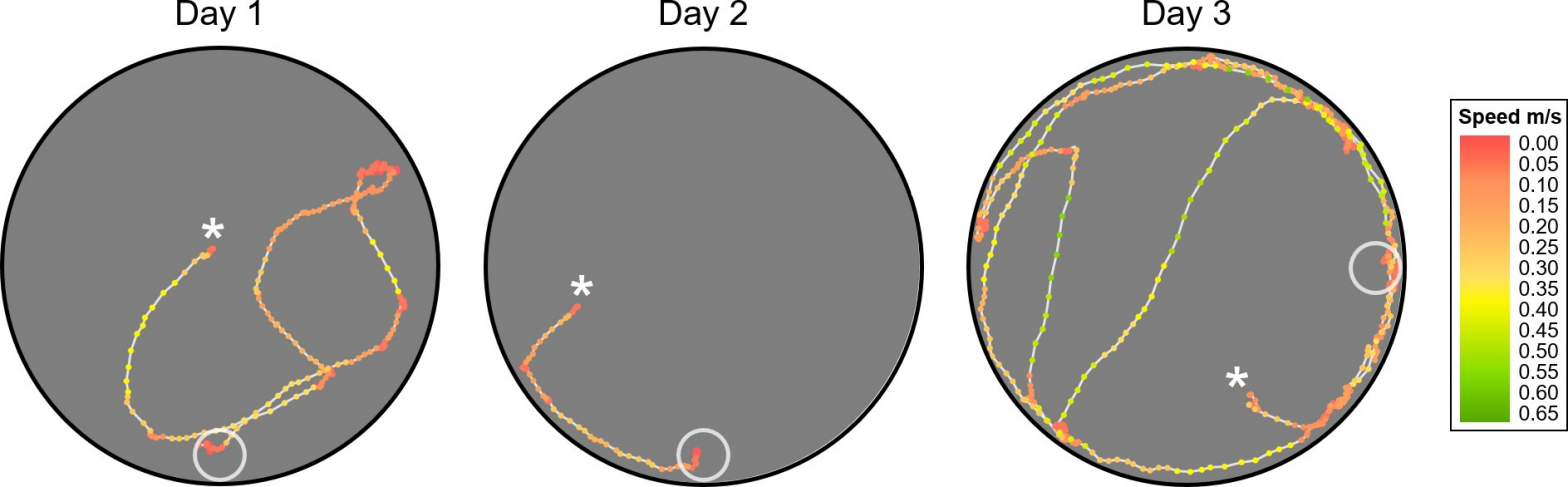
Path traces of a rat in the SD group over days. The seventh trial on day 1 (left), day 2 (center), and day 3 (after SD; right) for the same rat is shown. The marker position indicates the rat’s centroid position within the maze, and the average speed per 0.5 s in the maze is indicated by marker color, with red indicating slower speeds, yellow indicating intermediate speeds, and green indicating faster speeds (see legend). The large outer circle indicates the arena’s perimeter wall, with the rat’s approximate entry and exit locations for each trial indicated with a white circle and a white asterisk, respectively.

Comparison of the path traces of trial 7 on days 1 and 2 for a representative rat in the SD group showed that the rat’s path distance markedly improved across days and that the percent of time moving toward the target was much greater on day 2 (approximately 96%) (Fig 8). However, on day 3, following SD, the rat was much less efficient at locating the unmarked target, as evidenced by substantial increases in both trial time and path distance. The path trace itself also shows evidence of increased thigmotaxis behavior and a decrease in time spent moving toward the target, which may be an indirect measure of the ability of the rat to effectively use performance feedback to monitor and adjust path direction with respect to the unmarked target.

## 5. Discussion

Motivating rodents to perform cognitive task paradigms often relies on the application of appetitive or aversive stimuli. Here we investigated whether the use of floor vibration as a motivational stimulus may overcome potential confounds associated with stimuli used in other task paradigms. In a series of three experiments, we found that rats exhibit behavioral preference for an environment with floor vibrations over water, foot shock, and bright light environments. Furthermore, we found that physiological stress, as measured by corticosterone, was increased after exposure to floor vibrations relative to no exposure to motivational stimuli, but not as much as after forced swimming. Moreover, we showed that the VAST, a novel open-field task in which gradient floor vibration provides motivation and feedback, is rapidly and robustly learned by Long Evans rats (aged 8−12 weeks), and that 6 h SD causes significant performance impairment on the task despite relatively small sample sizes.

Our findings are consistent with previous research showing that rodents are naturally sensitive to vibrations [10]. Extended rat body vibration exposure (240 min) has been reported to increase circulating glucocorticoid levels and affect brain noradrenaline and serotonin levels [13, 14]. In the present application of the VAST, rats were exposed to vibration for approximately 5 minutes on average, and even rats with a high number of failed trials experienced no more than 10 minutes of vibration. This relatively limited exposure duration coupled with our habituation protocol likely minimized any systemic biochemical responses to the floor vibrations.

The use of orbital shakers to examine “earthquake” stress has been found to produce consistent negative biochemical and cardiovascular outcomes in rats, with repeated shaking for 5 min over 10 days resulting in increased plasma epinephrine, norepinephrine, and corticosterone concentrations [15]. The shaker vibration frequency used in these orbital shaker studies was 140 Hz, while the maximum vibration frequency used in our VAST experiments was 70 Hz. Moreover, the orbital shaker had a side-to-side 2−3 cm floor orbit, and the authors reported that the rodents struggled to maintain balance [15]. In the VAST, the rats navigated the open field with comparative ease since the vibration effects on floor displacement are minimal due to the anchored rotary motors that spin in the vertical axis and the equidistant position of these motors to the center of the arena.

In addition to serving as a motivational stimulus with only modest induction of stress, the floor vibrations in the VAST provide moment-to-moment feedback on the animal’s progress toward locating the target destination. Commonly used rodent task paradigms with motivational stimuli do not explicitly provide directional information or feedback. Therefore, the novel directional component of the VAST task may be useful for assessing the biological substrates of more complex decision-making and the effects of SD thereon. Our results showed that multiple performance metrics of the VAST are sensitive to 6 h SD in rats, which suggests that performance of the task relies on cognitive functions and neural pathways that are altered by sleep loss. Specifically, the VAST probes the rat’s ability to detect, filter, and retain pertinent cues to respond to, interact with, and adapt to the environment.

While it is possible that SD degraded performance on the VAST through other aspects of cognition, such as the ability to sustain attention [2, 16], our results suggest that 6 h SD likely impaired decision making on the VAST. In our study, SD caused a modest decrease in the percent of time moving toward the target out of total time moving, which is more likely to reflect reduced ability to correctly utilize directional information provided by vibration feedback than reduced ability to sustain attention. Furthermore, the percent of time still, which could be a marker of deficits in sustained attention (e.g., greater intrusion of microsleeps) was not found to differ between well-rested and sleep-deprived animals. Rather, our results suggest that SD impacts VAST performance via deterioration of cognitive processes related to decision making and the ability to use performance feedback.

Performance feedback is often used in tests of human cognition in the context of impairment due to SD, such as paired associates learning [17, 18], task rule or goal switching [19, 20], or cognitive flexibility [21–23]. Sleep loss produces considerable deficits in decision making that requires the use of outcome feedback to monitor and adapt one’s own performance [24], although the underlying mechanisms are subject to investigation and debate [25–27]. In the VAST, the observed effects of SD may have been caused by diminished potential of the vibration feedback to drive behavior. In humans, SD has been found to reduce the salience of information signaling a need for cognitive flexibility—a phenomenon termed “feedback blunting” [24]. An explanation for the rat VAST results in terms of diminished motivational drive from feedback during SD would thus be consistent with a possible explanation for loss of cognitive flexibility in humans due to SD. Establishing the VAST as a new animal model to facilitate such inquiry may help to further advance this area of research. The VAST could also be modified to accommodate the study of additional aspects of cognitive performance, such as reversal learning.

A comparison of VAST performance in rats versus mice [9] revealed noteworthy similarities and differences between the two paradigms. In the mouse VAST, performance of the task also improved over two days and was negatively impacted by subsequent SD. However, the metrics that changed across days and in response to SD differed for the mouse and rat versions of the task. Well-rested mice performed the task with lower success rates on days 1 and 2 (approximately 85%) versus the well-rested rats in the present study, which exhibited near-perfect success. Moreover, success rate was severely impacted by SD in mice, but much less so in rats. For the Long Evans rats studied here, path distance was most sensitive to learning within- and between-days, as well as to impairment from 6 h SD. In contrast, path distance remained stable over days and following SD in mice, which showed more substantial effects for time to target, speed, and time still [9]. These distinctions could reflect between-species differences in salience of the mechanical stimulation, search strategies, or variations in the experimental protocols used for the mouse and rat versions of the task (e.g., number of trials per day). Nonetheless, each of these studies establish the VAST, and its use of floor vibration as a motivational and directional stimulus, as a novel tool for the assessment of cognitive performance in rodents.

In conclusion, the VAST is a novel, versatile, goal-oriented rodent performance task that is poised to address outstanding questions in neuroscience and sleep research, such as the underlying mechanisms of SD-related errors in feedback-driven decision making. Here, we showed that VAST performance in rats stabilizes by day 2 of testing (with 9 trials each day), indicating that the task is learned quickly relative to other commonly used tasks for assessing cognitive performance (e.g., operant learning paradigms). Furthermore, we demonstrated medium to large effects of SD on multiple performance metrics, with relatively small sample sizes (n = 7 in the SD and CONT groups). As such, the VAST overcomes experimental hurdles associated with long training periods to achieve stable performance and large sample size requirements for statistical testing. The VAST also avoids technical issues associated with the Morris water maze [3], including difficulties managing electrophysiological recordings in the presence of water and potential confounds from thermoregulation challenges for the animal. Finally, in future studies it will be possible to modify the VAST to accommodate the study of other cognitive processes of interest, such as reversal learning [23, 24], and deficits in rodent models of neurodegenerative disease [28, 29] through examination of the associated behavioral correlates of compromised sensory detection, feedback interpretation, and motor responding.

## Supporting information

S1 Table Place preference raw dataset. (PDF)

S2 Table Corticosterone raw dataset. (PDF)

S3 Table A. VAST primary raw dataset. B. VAST secondary raw dataset.
(PDF)
