# Supplementary material for: Floor vibrations for motivation and feedback in the rat vibration actuating search task
Source: PLoS One. 2021 Sep 27;16(9):e0257980. doi: 10.1371/journal.pone.0257980 (PMC8475976; doi:10.1371/journal.pone.0257980)
Supplement: S1 Table — (PDF) [file pone.0257980.s001.pdf]

ID	Condition	Duration (s) - Vibration side	Duration (s) - Alternative side	Duration (s) - Total	Duration (%) - Vibration side	Duration (%) - Alternative side	Dwell duration per entrance (s) - Vibration side	Dwell duration per entrance (s) - Alternative side	Number of entrances - Vibration side	Number of entrances - Alternative side
5250	Light	167	73	240	69.58	30.42	83.50	36.50	6	6
5251	Light	167	73	240	69.58	30.42	83.50	36.50	4	3.5
5252	Light	124	116	240	51.67	48.33	62.00	58.00	7	7
5253	Light	132	109	241	54.77	45.23	66.00	54.50	5	5.5
5241	Shock	193	34	227	85.02	14.98	96.50	17.00	1.5	1
5242	Shock	212	15	227	93.39	6.61	106.00	7.50	2	1.5
5244	Shock	200	23	223	89.69	10.31	100.00	11.50	2.5	2
5237	Water	230	1	231	99.57	0.43	115.00	0.50	0.5	0
5238	Water	226	1	227	99.56	0.44	113.00	0.50	0.5	0
5239	Water	216	17	233	92.70	7.30	108.00	8.50	0.5	0
5240	Water	225	11	236	95.34	4.66	112.50	5.50	0.5	0
