# Supplementary material for: Floor vibrations for motivation and feedback in the rat vibration actuating search task
Source: PLoS One. 2021 Sep 27;16(9):e0257980. doi: 10.1371/journal.pone.0257980 (PMC8475976; doi:10.1371/journal.pone.0257980)
Supplement: S2 Table — (PDF) [file pone.0257980.s002.pdf]

ID	Condition	Plate	Corticosterone (ng/ml)
301	TOD	1	11.9628
302	TOD	1	16.9068
303	TOD	1	21.7784
304	TOD	1	11.4325
338	TOD	1	32.2305
339	TOD	1	35.6223
340	TOD	1	23.3434
341	TOD	1	37.1553
313	Vibration	1	38.6191
314	Vibration	1	36.4624
315	Vibration	1	27.3094
316	Vibration	1	38.7457
336	Vibration	1	28.082
337	Vibration	1	29.9812
347	Vibration	1	38.8372
348	Vibration	1	31.5809
349	Vibration	1	37.5734
317	Water	1	32.2305
318	Water	1	38.301
319	Water	1	48.0412
320	Water	1	35.7449
342	Water	1	35.3425
343	Water	1	43.8227
344	Water	1	41.6851
345	Water	1	42.1654
308	Light	2	48.8658
309	Light	2	42.5181
310	Light	2	35.5718
322	Light	2	31.4348
323	Light	2	37.4387
324	Light	2	36.0947
334	Light	2	18.9931
335	Light	2	37.4507
305	Shock	2	15.677
306	Shock	2	21.9765
307	Shock	2	23.2981
311	Shock	2	31.6358
312	Shock	2	33.2514
331	Shock	2	23.9002
332	Shock	2	24.8893
333	Shock	2	38.5475
313	Vibration	2	33.432
314	Vibration	2	31.193
315	Vibration	2	26.2165
316	Vibration	2	33.9235
336	Vibration	2	23.6425
337	Vibration	2	25.9927
347	Vibration	2	37.5778
348	Vibration	2	30.0756
349	Vibration	2	33.1943
