# Supplementary material for: Floor vibrations for motivation and feedback in the rat vibration actuating search task
Source: PLoS One. 2021 Sep 27;16(9):e0257980. doi: 10.1371/journal.pone.0257980 (PMC8475976; doi:10.1371/journal.pone.0257980)
Supplement: S3 Table — A. VAST primary raw dataset. B. VAST secondary raw dataset. (PDF) [file pone.0257980.s003.pdf]

ID	Day	Cohort	Group	Success (%)	Time (s)	Distance (m)	AUC (m•s)	Speed (m/s)	Time still (s)	Time moving towards the target (%)	Time still (%)
148	1	2	CONT	100.00	20.20	3.12	46.85	0.17	11.79	71.21	58.35
160	1	2	CONT	100.00	24.32	2.20	44.63	0.13	19.10	84.19	78.53
161	1	2	CONT	88.89	21.06	2.87	113.15	0.18	14.14	83.52	67.17
162	1	2	CONT	100.00	20.23	2.06	25.90	0.12	15.41	83.63	76.16
1001	1	5	CONT	100.00	25.36	3.00	52.78	0.13	18.22	69.76	71.87
1002	1	5	CONT	100.00	44.80	5.94	217.56	0.14	33.19	65.83	74.08
1003	1	5	CONT	88.89	41.37	4.67	176.30	0.15	30.83	65.29	74.54
168	1	3	SD	100.00	34.43	4.35	105.87	0.16	23.53	68.44	68.34
171	1	3	SD	100.00	37.78	4.64	211.92	0.15	25.56	77.09	67.65
172	1	3	SD	77.78	52.32	9.37	387.14	0.20	28.74	61.54	54.94
169	1	4	SD	100.00	38.92	5.54	156.70	0.13	25.40	65.86	65.26
173	1	4	SD	100.00	25.30	4.22	100.05	0.18	14.30	74.28	56.52
174	1	4	SD	100.00	40.10	4.35	106.32	0.10	28.88	70.56	72.01
175	1	4	SD	100.00	35.23	6.09	189.53	0.19	21.73	58.13	61.68
148	2	2	CONT	100.00	15.26	2.02	26.61	0.17	10.44	80.15	68.46
160	2	2	CONT	100.00	19.73	1.92	25.19	0.12	14.80	81.92	75.00
161	2	2	CONT	100.00	31.55	3.70	78.07	0.11	23.28	69.03	73.79
162	2	2	CONT	100.00	38.09	4.18	115.18	0.11	27.82	70.21	73.04
1001	2	5	CONT	100.00	27.13	2.75	64.30	0.11	21.36	78.31	78.71
1002	2	5	CONT	100.00	30.14	4.46	127.24	0.19	19.56	69.36	64.87
1003	2	5	CONT	100.00	17.60	3.41	47.14	0.21	9.19	64.42	52.21
168	2	3	SD	100.00	14.70	2.00	40.12	0.22	9.71	82.99	66.06
171	2	3	SD	100.00	41.37	4.47	162.52	0.12	30.18	68.51	72.95
172	2	3	SD	88.89	44.81	5.42	163.82	0.14	32.32	66.87	72.12
169	2	4	SD	88.89	33.01	3.44	101.01	0.13	23.83	75.06	72.20
173	2	4	SD	88.89	28.89	3.01	76.79	0.14	21.44	76.08	74.23
174	2	4	SD	100.00	42.87	4.76	125.42	0.11	30.38	70.53	70.86
175	2	4	SD	100.00	19.41	3.04	39.84	0.17	12.29	71.95	63.31
148	3	2	CONT	100.00	16.38	2.59	36.19	0.17	9.77	68.64	59.64
160	3	2	CONT	100.00	26.31	2.50	43.53	0.10	20.20	79.75	76.77
161	3	2	CONT	100.00	17.54	2.35	21.94	0.14	11.94	69.29	68.08
162	3	2	CONT	88.89	53.87	5.27	241.05	0.10	41.03	69.79	76.17
1001	3	5	CONT	100.00	29.17	3.19	78.81	0.12	21.67	77.53	74.28
1002	3	5	CONT	100.00	21.98	4.22	62.19	0.21	12.17	63.18	55.36
1003	3	5	CONT	100.00	14.03	2.98	27.90	0.25	6.76	72.06	48.14
168	3	3	SD	88.89	67.01	5.26	198.21	0.09	54.97	64.47	82.03
171	3	3	SD	100.00	22.19	3.51	46.31	0.17	11.87	69.35	53.48
172	3	3	SD	100.00	36.75	7.39	183.19	0.20	17.24	62.56	46.93
169	3	4	SD	88.89	41.64	6.56	200.86	0.16	23.57	65.30	56.60
173	3	4	SD	88.89	54.73	6.44	239.48	0.13	38.74	66.09	70.79
174	3	4	SD	77.78	61.88	5.89	240.63	0.10	47.74	67.25	77.16
175	3	4	SD	100.00	22.14	4.12	68.06	0.21	12.61	66.92	56.95

ID	Day	Trial block	Cohort	Group	Time (s)	Distance (m)	AUC (m•s)
148	1	1	2	CONT	18.77	3.30	40.44
160	1	1	2	CONT	25.50	3.35	60.84
161	1	1	2	CONT	9.67	1.58	7.13
162	1	1	2	CONT	13.00	1.32	8.21
1001	1	1	5	CONT	31.03	4.02	90.22
1002	1	1	5	CONT	41.47	6.73	306.01
1003	1	1	5	CONT	48.57	6.07	228.69
168	1	1	3	SD	36.93	3.96	157.18
171	1	1	3	SD	73.60	8.95	555.15
172	1	1	3	SD	58.27	9.65	390.54
169	1	1	4	SD	43.50	8.55	282.46
173	1	1	4	SD	41.43	8.12	230.52
174	1	1	4	SD	43.50	4.94	95.56
175	1	1	4	SD	46.70	9.62	398.89
148	1	2	2	CONT	15.91	2.35	24.41
160	1	2	2	CONT	44.10	2.62	72.27
161	1	2	2	CONT	45.50	5.79	326.41
162	1	2	2	CONT	27.97	2.68	34.15
1001	1	2	5	CONT	22.17	2.63	39.82
1002	1	2	5	CONT	37.07	4.98	116.15
1003	1	2	5	CONT	32.13	4.28	78.36
168	1	2	3	SD	35.80	5.63	105.93
171	1	2	3	SD	29.83	3.85	76.08
172	1	2	3	SD	71.50	12.61	640.85
169	1	2	4	SD	43.53	5.19	151.09
173	1	2	4	SD	7.23	1.42	6.33
174	1	2	4	SD	46.93	5.88	196.66
175	1	2	4	SD	23.20	4.28	80.83
148	1	3	2	CONT	25.93	3.70	75.71
160	1	3	2	CONT	3.37	0.64	0.78
161	1	3	2	CONT	8.00	1.24	5.92
162	1	3	2	CONT	19.73	2.18	35.33
1001	1	3	5	CONT	22.87	2.34	28.31
1002	1	3	5	CONT	55.87	6.11	230.52
1003	1	3	5	CONT	43.40	3.65	221.86
168	1	3	3	SD	30.57	3.48	54.49
171	1	3	3	SD	9.90	1.12	4.54
172	1	3	3	SD	27.20	5.86	130.02
169	1	3	4	SD	29.73	2.90	36.55
173	1	3	4	SD	27.24	3.12	63.31
174	1	3	4	SD	29.87	2.24	26.73
175	1	3	4	SD	35.80	4.36	88.86
148	2	1	2	CONT	23.10	2.73	50.10
160	2	1	2	CONT	9.10	1.37	10.92
161	2	1	2	CONT	43.27	4.42	114.26
162	2	1	2	CONT	44.40	4.35	96.27
1001	2	1	5	CONT	17.37	2.74	45.00
1002	2	1	5	CONT	44.60	6.30	220.91
1003	2	1	5	CONT	12.30	2.21	14.32
168	2	1	3	SD	31.93	3.27	112.42
171	2	1	3	SD	38.13	4.12	124.94
172	2	1	3	SD	35.37	5.24	116.55
169	2	1	4	SD	55.07	5.25	227.51
173	2	1	4	SD	26.77	3.00	51.75
174	2	1	4	SD	63.87	7.02	222.32
175	2	1	4	SD	24.60	3.60	46.41
148	2	2	2	CONT	17.93	2.44	26.96
160	2	2	2	CONT	28.40	2.68	45.10
161	2	2	2	CONT	28.50	3.32	66.63
162	2	2	2	CONT	42.94	4.96	175.56
1001	2	2	5	CONT	12.40	0.88	6.07
1002	2	2	5	CONT	34.20	5.37	147.65
1003	2	2	5	CONT	15.43	3.55	27.69
168	2	2	3	SD	5.03	1.05	1.49
171	2	2	3	SD	56.77	5.47	280.54
172	2	2	3	SD	30.04	3.72	98.47
169	2	2	4	SD	19.54	2.41	27.14
173	2	2	4	SD	47.47	4.30	169.39
174	2	2	4	SD	32.53	3.17	63.09
175	2	2	4	SD	21.77	3.28	56.36
148	2	3	2	CONT	4.73	0.88	2.78
160	2	3	2	CONT	21.70	1.69	19.56
161	2	3	2	CONT	22.87	3.37	53.32
162	2	3	2	CONT	26.93	3.24	73.71
1001	2	3	5	CONT	51.63	4.62	141.81
1002	2	3	5	CONT	11.63	1.69	13.16
1003	2	3	5	CONT	25.07	4.48	99.41
168	2	3	3	SD	7.13	1.67	6.45
171	2	3	3	SD	29.20	3.81	82.07
172	2	3	3	SD	69.03	7.30	276.43
169	2	3	4	SD	24.43	2.66	48.39
173	2	3	4	SD	12.43	1.74	9.23
174	2	3	4	SD	32.20	4.07	90.85
175	2	3	4	SD	11.87	2.23	16.76
148	3	1	2	CONT	28.10	4.35	88.50
160	3	1	2	CONT	16.17	1.99	15.97
161	3	1	2	CONT	23.77	3.23	37.18
162	3	1	2	CONT	17.33	1.96	18.99
1001	3	1	5	CONT	26.23	3.26	89.31
1002	3	1	5	CONT	23.83	4.41	92.70
1003	3	1	5	CONT	5.90	1.63	5.45
168	3	1	3	SD	34.00	3.53	50.83
171	3	1	3	SD	21.90	4.21	55.50
172	3	1	3	SD	31.27	6.66	147.15
169	3	1	4	SD	21.27	3.96	41.89
173	3	1	4	SD	68.17	8.14	374.17
174	3	1	4	SD	66.23	6.57	297.00
175	3	1	4	SD	34.57	6.27	145.81
148	3	2	2	CONT	8.07	1.21	6.02
160	3	2	2	CONT	39.73	3.76	92.55
161	3	2	2	CONT	10.40	1.91	10.91
162	3	2	2	CONT	66.77	6.82	338.49
1001	3	2	5	CONT	17.70	1.97	16.92
1002	3	2	5	CONT	25.67	4.90	62.84
1003	3	2	5	CONT	23.04	3.84	49.97
168	3	2	3	SD	79.73	7.34	349.77
171	3	2	3	SD	25.73	3.41	41.90
172	3	2	3	SD	36.90	8.02	201.64
169	3	2	4	SD	39.61	5.96	153.06
173	3	2	4	SD	65.43	7.32	258.55
174	3	2	4	SD	70.93	6.94	313.50
175	3	2	4	SD	17.47	2.99	34.51
148	3	3	2	CONT	12.97	2.19	14.04
160	3	3	2	CONT	23.03	1.74	22.07
161	3	3	2	CONT	18.47	1.89	17.72
162	3	3	2	CONT	77.50	7.04	365.66
1001	3	3	5	CONT	43.57	4.34	130.20
1002	3	3	5	CONT	16.43	3.34	31.03
1003	3	3	5	CONT	13.17	3.47	28.30
168	3	3	3	SD	87.30	4.89	194.04
171	3	3	3	SD	18.93	2.92	41.52
172	3	3	3	SD	42.07	7.48	200.79
169	3	3	4	SD	64.03	9.75	407.61
173	3	3	4	SD	30.60	3.87	85.72
174	3	3	4	SD	48.47	4.15	111.39
175	3	3	4	SD	14.40	3.10	23.85
